# Acute SARS-CoV-2 Infection and Incidence and Outcomes of Out-of-Hospital Cardiac Arrest

**DOI:** 10.1001/jamanetworkopen.2023.36992

**Published:** 2023-10-06

**Authors:** Jennifer Z. Liu, Catherine R. Counts, Christopher J. Drucker, Jamie M. Emert, David L. Murphy, Leilani Schwarcz, Peter J. Kudenchuk, Michael R. Sayre, Thomas D. Rea

**Affiliations:** 1 Emergency Medical Services Division, Public Health–Seattle & King County, Seattle, Washington; 2 Seattle Fire Department, Seattle, Washington; 3 Department of Emergency Medicine, University of Washington, Seattle; 4 Division of Cardiology, University of Washington, Seattle; 5 Department of Medicine, University of Washington, Seattle

## Abstract

**Importance:**

Little is known about how COVID-19 affects the incidence or outcomes of out-of-hospital cardiac arrest (OHCA), and it is possible that more generalized factors beyond SARS-CoV-2 infection are primarily responsible for changes in OHCA incidence and outcome.

**Objective:**

To assess whether COVID-19 is associated with OHCA incidence and outcomes.

**Design, Setting, and Participants:**

This retrospective cohort study was conducted in Seattle and King County, Washington. Participants included persons aged 18 years or older with nontraumatic OHCA attended by emergency medical services (EMS) between January 1, 2018, and December 31, 2021. Data analysis was performed from November 2022 to March 2023.

**Exposures:**

Prepandemic (2018-2019) and pandemic (2020-2021) periods and SARS-CoV-2 infection.

**Main Outcomes and Measures:**

The primary outcomes were OHCA incidence and patient outcomes (ie, survival to hospital discharge). Mediation analysis was used to determine the percentage change in OHCA incidence and outcomes between prepandemic and pandemic periods that was attributable to acute SARS-CoV-2 infection vs conventional Utstein elements related to OHCA circumstances (ie, witness status and OHCA location) and resuscitation care (ie, bystander cardiopulmonary resuscitation, early defibrillation, and EMS response intervals).

**Results:**

There were a total of 13 081 patients with OHCA (7102 dead upon EMS arrival and 5979 EMS treated). Among EMS-treated patients, the median (IQR) age was 64.0 (51.0-75.0) years, 3864 (64.6%) were male, and 1027 (17.2%) survived to hospital discharge. The total number of patients with OHCA increased by 19.0% (from 5963 in the prepandemic period to 7118 in the pandemic period), corresponding to an incidence increase from 168.8 to 195.3 events per 100 000 person-years. Of EMS-treated patients with OHCA during the pandemic period, 194 (6.2%) were acutely infected with SARS-CoV-2 compared with 7 of 191 EMS-attended but untreated patients with OHCA (3.7%). In time-series correlation analysis, there was a positive correlation between community SARS-CoV-2 incidence and overall OHCA incidence (*r* = 0.27; *P* = .01), as well as OHCA incidence with acute SARS-CoV-2 infection (*r* = 0.43; *P* < .001). The survival rate during the pandemic period was lower than that in the prepandemic period (483 patients [15.4%] vs 544 patients [19.2%]). During the pandemic, those with OHCA and acute SARS-CoV-2 infection had lower likelihood of survival compared with those without acute infection (12 patients [6.2%] vs 471 patients [16.0%]). SARS-CoV-2 infection itself accounted for 18.5% of the pandemic survival decline, whereas Utstein elements mediated 68.2% of the survival decline.

**Conclusions and Relevance:**

In this cohort study of COVID-19 and OHCA, a substantial proportion of the higher OHCA incidence and lower survival during the pandemic was not directly due to SARS-CoV-2 infection but indirect factors that challenged OHCA prevention and treatment.

## Key Points

**Question:**

What proportion of the change in out-of-hospital cardiac arrest (OHCA) incidence and outcomes between the prepandemic and pandemic periods was associated with patient-specific acute SARS-CoV-2 infection?

**Findings:**

In this cohort study of 13 081 patients, the incidence of OHCA increased by 19.0% during the pandemic, even though only 6.2% of emergency medical services (EMS)–treated patients and 3.7% of EMS-attended but untreated patients during the pandemic period were classified with acute SARS-CoV-2 infection. OHCA survival decreased from 19.2% to 15.4%; acute SARS-CoV-2 infection accounted for 18.5% of this decrease, whereas more generalizable characteristics related to OHCA circumstances and resuscitation care mediated 68.2% of the decrease.

**Meaning:**

The findings underscore the adverse public health consequences resulting from indirect effects of the COVID-19 pandemic.

## Teaser

This cohort study assesses the association of acute SARS-CoV-2 infection with the incidence and outcomes of out-of-hospital cardiac arrest in Seattle and King County, Washington.

## Introduction

During the COVID-19 pandemic, the incidence of out-of-hospital cardiac arrest (OHCA) increased.^1,2,3,4^ Timely and expert resuscitation was also challenged.^5,6,7,8,9^ Taken together, the incidence increase and resuscitation challenge have resulted in a greater global public health toll.^10,11^

Little is known about how COVID-19 affected OHCA incidence and outcomes. The increase in OHCA incidence may be directly attributed to complications of SARS-CoV-2 infection. Alternatively, the pandemic may affect OHCA incidence via indirect factors (eg, persons delaying preventative care or being reticent to activate 911 for prodromal symptoms to avoid hospital evaluation). Regarding resuscitation, SARS-CoV-2 infection may render patients with OHCA more refractory to treatment.^12,13^ Alternatively, the pandemic may have produced more generalized challenges independent of patient-specific infection, such as altering societal behaviors or clinical practice, thereby changing OHCA circumstances or hindering rescuer efforts.

A better understanding of the potential factors by which COVID-19 may have influenced OHCA incidence and patient outcomes has important implications for how to best prevent and treat pandemic-related health challenges, specifically OHCA. We evaluated the association of community SARS-CoV-2 incidence with overall and COVID-19–specific OHCA incidence and overall and COVID-19–specific OHCA outcomes to better understand how COVID-19 could influence OHCA incidence and outcome. We hypothesized that indirect effects of the pandemic, rather than patient-specific SARS-CoV-2 infection, were primarily responsible for changes in OHCA incidence and outcome.

## Methods

### Study Design, Population, and System

We conducted a retrospective cohort study assessing COVID-19 and nontraumatic OHCA among persons aged 18 years or older attended by emergency medical services (EMS) in Seattle and King County, Washington, between January 1, 2018, and December 31, 2021. The years 2018 and 2019 provided a control period to compare and contrast incidence and outcome of pandemic-related OHCA. Surveillance of community SARS-CoV-2 incidence was also incorporated to understand how changes in community incidence may be associated with OHCA incidence. The study was approved by the review boards of the University of Washington and Public Health–Seattle & King County and used Strengthening the Reporting of Observational Studies in Epidemiology (STROBE) reporting guideline for observational research.^14^ Informed consent was waived because the investigation was considered minimal risk, in accordance with 45 CFR §46.

King County is a metropolitan region with a population of 2.3 million residing in urban, suburban, and rural areas. Individuals activate EMS response by calling 911 and contacting a telecommunicator, who uses questions about consciousness and breathing to identify suspected OHCA and to coach layperson cardiopulmonary resuscitation (CPR). King County EMS (KCEMS) response is 2-tiered. The first tier comprises firefighter emergency medical technicians trained in CPR and automated external defibrillator (AED) use. The second tier comprises paramedics, whose scope of practice includes electrocardiogram rhythm interpretation, manual defibrillation, parenteral drug administration, and advanced airway management. Patients achieving return of spontaneous circulation are transported to 1 of 12 hospitals, each equipped with intensive care unit and around-the-clock coronary catheterization facilities.

Throughout the pandemic, KCEMS followed infection prevention and control practices recommended by the Centers for Disease Control and Prevention, which included protocols for personal protective equipment and screening and care of patients at risk for acute COVID-19. Clinical protocols for resuscitation did not change in response to the pandemic, except for the addition of masks and gowns for personal protective equipment and the application of high-efficiency particulate air filters to ventilation bags.^15,16,17^

### Data Sources and Definitions

#### OHCA Utstein Elements

KCEMS maintains a registry of OHCA organized according to the Utstein template.^18^ Information about patient, circumstance, treatment, and outcome is systematically abstracted from dispatch audio recordings, defibrillator electronic data, prehospital and hospital records, death certificates, and medical examiner reports. Collectively, these measures are referred to as *Utstein elements*.^18^

#### Patient COVID-19 Status

KCEMS is administered by Public Health–Seattle & King County, enabling direct engagement to undertake COVID-19 surveillance to determine EMS involvement among patients with COVID-19.^17,19^ We linked EMS records with Washington Disease Reporting System (WDRS). WDRS contains the name, date of birth, test dates, and results for all individuals who have been tested for SARS-CoV-2 within Washington. The link between WDRS and EMS record was performed via a multiple-step deterministic algorithm using the patient’s first and last name, date of birth, and age, followed by review to confirm potential links. For patients without a link or those who had test results outside a predetermined infection window, we classified COVID-19 status through review of death certificates, EMS patient care reports, and hospital records (when available) using a previously reported algorithm with good interreviewer reliability.^20^ We classified a patient as being infected with SARS-CoV-2 (acute COVID-19) if the positive test was between 14 days before and 7 days after the OHCA date. Sensitivity analyses extended COVID-19 classification to 28 days before and 7 days after the OHCA. We undertook COVID-19 classification for all EMS-treated patients and a random 5% sample of persons who were attended by EMS but were determined to be dead on arrival (DOA), whereby resuscitation was not initiated because of evidence of irreversible death (ie, rigor mortis or lividity). SARS-CoV-2 testing during the acute infection window was available for 49.9% of EMS-treated patients with OHCA and 38.7% of those who were DOA (eFigures 1 and 2 in Supplement 1). Public Health–Seattle & King County, in collaboration with Washington State Department of Health, undertakes community COVID-19 surveillance.^21^

### Outcomes

The study evaluated OHCA incidence and outcomes related to the pandemic period and specifically to COVID-19 status. Hence, we determined OHCA incidence with and without SARS-CoV-2 infection and resuscitation outcomes. Resuscitation outcomes included hospital admission, survival to hospital discharge, and favorable neurological survival (cerebral performance category 1 or 2).

### Statistical Analysis

Descriptive statistics were calculated to compare characteristics according to time period (2018-2019 vs 2020-2021) and COVID-19 status using the *t* test, Mann-Whitney *U* test, χ^2^ test, and Fisher exact test. COVID-19 and OHCA incidences were defined as events per 100 000 person-years among the adult population (aged ≥18 years). We calculated weekly and 4-week rolling means of community COVID-19 incidence, OHCA incidence overall, and OHCA incidence with acute COVID-19. Cross-correlation was conducted to determine the temporal association of community COVID-19 with OHCA incidence overall and the association of OHCA with SARS-CoV-2 infection.^22^ A priori subgroup analyses were restricted to persons aged 65 years or older given the potential that older persons may be specifically vulnerable to COVID-19–related OHCA. We also evaluated 2020 and 2021 separately given the potential influence of vaccination and/or distinct COVID-19 variants.

We compared resuscitation outcomes for the prepandemic vs pandemic periods using logistic regression. We initially conducted unadjusted analyses to determine the odds ratio (OR) of resuscitation outcomes associated with time period (prepandemic as reference). To estimate the direct contribution of COVID-19 to survival differences, we removed patients with acute SARS-CoV-2 infection and repeated the regression to compare percentage change in β coefficients using the following formula: 1 – (β for patients without COVID-19 / β for all patients).^23,24^

We then sought to understand how Utstein elements mediated resuscitation outcomes among patients without acute infection. Mediating variables were selected a priori on the basis of published evidence through a directed acyclic graph. Referred to as the *Utstein model*, the cause of OHCA, arrest location, witness status, bystander CPR, non-EMS automated external defibrillator application, interval from 911 call to EMS at the patient’s side, and OHCA before EMS arrival were included (eFigure 3 in Supplement 1). We assessed whether the Utstein elements mediated change in pandemic period survival using the following formula: 1 – (β adjusted for Utstein elements / β unadjusted for patients without COVID-19). For sensitivity analysis, we incorporated patient age, sex, and initial rhythm into the Utstein model to assess whether the additions further mediated survival change. Analyses were repeated for the Utstein subgroup bystander-witnessed OHCA with a shockable initial rhythm (bystander-witnessed shockable OHCA). *P* < *.*05 was considered significant, and tests were 2-tailed. Analyses used R statistical software version 4.1.0 (R Project for Statistical Computing) and SAS statistical software version 9.4 (SAS Institute). Data analysis was performed from November 2022 to March 2023.

## Results

### Incidence

There were a total of 13 081 EMS-attended patients with OHCA, 7102 who were DOA and 5979 who received attempted resuscitation. Among 5979 EMS-treated patients with OHCA, the median (IQR) age was 64.0 (51.0-75.0) years, 3864 (64.6%) were male, 2326 events (38.9%) were bystander witnessed, 2331 (38.9%) were admitted to hospital, and 1027 (17.2%) survived to hospital discharge. Of the 5979 EMS-treated cases, 2837 occurred during prepandemic years and 3142 occurred during pandemic years, representing a 10.8% increase. We also observed a 27.2% increase in EMS-attended DOA patients, from 3126 during prepandemic years to 3976 during the pandemic period. The total number of EMS-attended patients with OHCA, both treated and untreated, increased by 19.0% from 5963 during the prepandemic period to 7118 during the pandemic period. Compared with the prepandemic period, incidence during the pandemic increased from 168.8 to 195.3 events per 100 000 person-years for all EMS-attended OHCAs, from 80.3 to 86.2 per 100 000 person-years for EMS-treated OHCAs, and from 88.5 to 109.1 per 100 000 person-years for EMS-attended but untreated (DOA) OHCAs. The increase in EMS-treated incidence during the pandemic period was also observed for men and women and for older (≥65 years) and younger adults (<65 years) (Table 1).

**Table 1.** OHCA Incidence Among Persons Aged 18 Years or Older^a^

Variable	Incidence rate/100 000 population (95% CI)	
	Prepandemic period (2018-2019)	Pandemic period (2020-2021)
All EMS-attended OHCA	168.8 (164.5-173.1)	195.3 (190.8-199.9)
EMS-attended untreated OHCA	88.5 (85.4-91.6)	109.1 (105.7-112.5)
EMS-treated OHCA		
Overall	80.3 (77.4-83.3)	86.2 (83.2-89.3)
Age group, y		
18-64	48.7 (46.2-51.3)	54.3 (51.7-57.0)
≥65	242.0 (229.4-255.0)	242.5 (230.4-255.1)
Sex		
Male	105.0 (100.3-109.9)	109.5 (104.8-114.4)
Female	55.5 (52.1-59.1)	62.7 (59.1-66.4)

Abbreviations: EMS, emergency medical services; OHCA, out-of-hospital cardiac arrest.

^a^ Population was based on estimates from Washington State Office of Financial Management and Census 2020 Public Law 94-171 data.

During the pandemic period, 194 of 5979 EMS-treated patients with OHCA (6.2%; 95% CI, 5.3%-7.0%) were classified as being acutely infected with SARS-CoV-2. Expansion of the infection window from 14 days before to 28 days before the OHCA event reclassified 2 patients as having acute SARS-CoV-2 infection. Among the random sample of DOA patients, 7 of 191 (3.7%; 95% CI, 1.0%-6.3%) were classified as acutely infected.

In correlation analysis, we observed a positive correlation between community COVID-19 incidence and EMS-treated OHCA incidence (*r* = 0.27; *P* = .01) during 2020 to 2021 (eFigure 4 in Supplement 1). When stratified by year, there was a significant positive correlation during 2020 (*r* = 0.39; *P* = .004), but not 2021 (*r* = −0.16; *P* = .25). The correlation was particularly evident for patients with OHCA and acute SARS-CoV-2 infection (*r* = 0.43; *P* < .001 for 2020-2021), even when stratified by year for 2020 (*r* = 0.59; *P* < .001) and 2021 (*r* = 0.36; *P* = .01). Similar correlations were observed when restricted to persons aged 65 years or older. OHCA incidence increased 0 to 2 weeks following an increase in community COVID-19 incidence.

### Resuscitation

We observed lower proportions of OHCA survival outcomes during the pandemic compared with the prepandemic period, including survival to hospital admission (1122 patients [35.7%] vs 1209 patients [42.6%]), survival to hospital discharge (483 patients [15.4%] vs 544 patients [19.2%]), and survival with favorable neurological status (432 patients [13.7%] vs 489 patients [17.2%]) (Table 2). Worse outcomes were also observed during the pandemic period for the Utstein subset (bystander-witnessed shockable OHCA), including hospital admission (282 patients [67.0%] vs 327 patients [76.4%]), survival to hospital discharge (176 patients [41.8%] vs 234 patients [54.7%]), and favorable neurological status (163 patients [38.7%] vs 216 patients [50.5%]) (eTable in Supplement 1). When we compared Utstein elements, we observed adverse trends during the pandemic for public location of OHCA, witness status, non-EMS automated external defibrillator use, and EMS response intervals (Table 2 and eTable in Supplement 1). Median age, proportion female, bystander CPR, and presenting arrest rhythm were not different between study periods. Targeted temperature management in hospital care was greater during the pandemic period (729 patients [65.0%] vs 717 patients [59.3%]). Although the proportion of patients undergoing coronary angiogram did not differ, a lower proportion underwent angiogram in the first 24 hours overall and for the Utstein subset during the pandemic (Table 2 and eTable in Supplement 1).

**Table 2.** Characteristics of EMS-Treated Patients With OHCA According to Time Period and SARS-CoV-2 Status

Characteristic	All patients, No. (%)			Pandemic period patients, No. (%)		
	Prepandemic period (n = 2837)^a^	Pandemic period (n = 3142)^a^	*P* value	No acute SARS-CoV-2 infection (n = 2948)	Acute SARS-CoV-2 infection (n = 194)	*P* value
Age, median (IQR), y	64.0 (52.0-75.0)	63.0 (51.0-75.0)	.16	64.0 (51.0-75.0)	59.0 (46.3-68.0)	<.01
Sex						
Male	1859 (65.5)	2005 (63.8)	.17	1876 (63.6)	129 (66.5)	.47
Female	978 (34.5)	1137 (36.2)		1072 (36.4)	65 (33.5)	
Cardiac cause	1686 (59.4)	1754 (55.8)	.01	1699 (57.6)	55 (28.4)	<.001
Location						
Home or other residence	1857 (65.5)	2339 (74.4)	<.001	2186 (74.2)	153 (78.9)	.26
Public (indoors or outdoors)	579 (20.4)	462 (14.7)		441 (15.0)	21 (10.8)	
Care facility (long-term care or clinic)	401 (14.1)	341 (10.9)		321 (10.9)	20 (10.3)	
OHCA before EMS arrival						
Before EMS	2442 (86.1)	2729 (86.9)	.40	2560 (86.8)	169 (87.1)	.99
After EMS	395 (13.9)	413 (13.1)		388 (13.2)	25 (12.9)	
Bystander witnessed^b^	1170 (47.9)	1156 (42.4)	<.001	1096 (42.8)	60 (35.5)	.07
Bystander cardiopulmonary resuscitation^b^	1180 (74.1)	2040 (74.8)	.62	1913 (74.7)	127 (75.1)	.98
Non-EMS automated external defibrillator application^b^	294 (12.0)	262 (9.6)		250 (9.8)	12 (7.1)	
Law enforcement	184 (7.5)	170 (6.2)	.02	161 (6.3)	9 (5.3)	.50
Layperson	110 (4.5)	92 (3.4)		89 (3.5)	3 (1.8)	
Initial shockable rhythm	637 (22.5)	665 (21.2)	.19	647 (21.9)	18 (9.3)	<.001
Time from 911 call to basic life support scene arrival, median (IQR), min	4.5 (3.6-5.8)	5.0 (3.9-6.4)	<.001	5.0 (3.9-6.4)	5.1 (4.2-6.4)	.22
Time from 911 call to advanced life support scene arrival, median (IQR), min	7.6 (5.8-10.0)	8.0 (6.2-10.3)	<.001	8.0 (6.2-10.3)	8.1 (6.3-10.4)	.63
Time from 911 call to EMS-at-patient’s-side interval, median (IQR), min	6.3 (4.8-7.9)	7.1 (5.4-8.9)	<.001	7.1 (5.4-8.9)	7.2 (5.8-8.8)	.55
Admitted to hospital	1209 (42.6)	1122 (35.7)	<.001	1071 (36.3)	51 (26.3)	.01
Hospital care^c^						
Targeted temperature management	717 (59.3)	729 (65.0)	.01	698 (65.2)	31 (60.8)	.58
Angiogram	429 (35.5)	368 (32.8)	.18	358 (33.4)	10 (19.6)	.06
Time from 911 call to angiogram, h						
<3	257 (59.9)	185 (50.3)	.01	181 (50.6)	4 (40.0)	.74
<6	281 (65.5)	212 (57.6)	.02	208 (58.1)	4 (40.0)	.30
<24	313 (73.0)	235 (63.9)	.004	229 (64.0)	6 (60.0)	.99
Withdrawal of life-sustaining treatment	557 (46.1)	530 (47.2)	.58	502 (46.9)	28 (54.9)	.35
Time from hospital admission to withdrawal of life-sustaining treatment, median (IQR), d	2.0 (1.0-5.0)	3.0 (1.0-6.0)	.05	3.0 (1.0-6.0)	3.5 (1.8-6.5)	.43
Survival						
Survived to hospital discharge	544 (19.2)	483 (15.4)	<.001	471 (16.0)	12 (6.2)	<.001
Favorable neurological survival (cerebral performance category 1-2)	489 (17.2)	432 (13.7)	<.001	420 (14.2)	12 (6.2)	.002

Abbreviations: EMS, emergency medical services; OHCA, out-of-hospital cardiac arrest.

^a^ The prepandemic period includes years 2018 and 2019, and the pandemic period includes years 2020 and 2021. Acute SARS-CoV-2 infection status is considered only during the pandemic period.

^b^ Restricted to OHCAs before EMS arrival.

^c^ Restricted to patients admitted to the hospital.

When we restricted analyses to the pandemic period and compared characteristics according to acute SARS-CoV-2 infection, those with acute infection were younger, less likely to present with shockable rhythm, less likely to have OHCA due to cardiac causes, and less likely to survive to hospital discharge (12 patients [6.2%] vs 471 patients [16.0%]) (Table 2). We did not observe a survival difference according to acute COVID-19 status when restricted to the Utstein subgroup (172 patients [42.0%] without COVID-19 vs 4 patients [36.4%] with COVID-19) (eTable in Supplement 1).

### Mediation

The unadjusted OR of survival to hospital discharge during the pandemic period compared with the prepandemic period was 0.77 (95% CI, 0.67-0.88) (Table 3). After the exclusion of 194 patients with acute SARS-CoV-2, the OR of survival was 0.80 (95% CI, 0.70-0.92), with acute infection contributing 18.5% of the overall lower odds. The addition of Utstein elements (Utstein model) produced a larger attenuation of pandemic outcome associations, with the resulting OR attenuated to 0.93 (95% CI, 0.80-1.08), mediating 68.2% of the survival decline between prepandemic and pandemic periods. When we used the intermediate outcome of hospital admission, the contributions of acute SARS-CoV-2 infection accounted for 10.3% of the decrease, whereas Utstein elements accounted for 26.9% of the decrease. Among bystander-witnessed shockable OHCA (Utstein group), acute SARS-CoV-2 infection did not contribute to the decrease in survival during the pandemic, whereas the Utstein elements accounted for 41.2% of survival decrease. Similar estimates were observed with additional adjustment for age, sex, and initial rhythm.

**Table 3.** Mediation Analysis: Association of Time Period With Resuscitation Outcomes Overall and Among the Utstein Group

Resuscitation outcomes	EMS-treated patients with OHCA (n = 5979)			Utstein group: bystander witnessed with initial shockable rhythm (n = 849)		
	OR (95% CI)	β coefficient	Change, %^a^	OR (95% CI)	β coefficient	Change, %^a^
Admission to hospital						
Total, unadjusted	0.75 (0.67-0.83)	−0.29	NA	0.62 (0.46-0.84)	−0.47	NA
Excluding acute SARS-CoV-2 infection, unadjusted	0.77 (0.69-0.85)	−0.26	10.3^b^	0.64 (0.47-0.87)	−0.44	6.4^b^
Excluding acute SARS-CoV-2 infection, with Utstein elements^c^	0.83 (0.74-0.93)	−0.19	26.9^d^	0.80 (0.58-1.10)	−0.22	50.0^d^
Survival to hospital discharge						
Total, unadjusted	0.77 (0.67-0.88)	−0.27	NA	0.60 (0.45-0.78)	−0.51	NA
Excluding acute SARS-CoV-2 infection, unadjusted	0.80 (0.70-0.92)	−0.22	18.5^b^	0.60 (0.46-0.79)	−0.51	0.0^b^
Excluding acute SARS-CoV-2 infection, with Utstein elements^c^	0.93 (0.80-1.08)	−0.07	68.2^d^	0.74 (0.55-0.99)	−0.30	41.2^d^
Favorable neurologic survival (cerebral performance category 1-2)						
Total, unadjusted	0.77 (0.66-0.88)	−0.27	NA	0.62 (0.47-0.81)	−0.48	NA
Excluding acute SARS-CoV-2 infection, unadjusted	0.80 (0.69-0.92)	−0.23	14.8^b^	0.62 (0.47-0.82)	−0.48	0.0^b^
Excluding acute SARS-CoV-2 infection, with Utstein elements^c^	0.92 (0.79-1.08)	−0.08	65.2^d^	0.76 (0.57-1.02)	−0.27	43.8^d^

Abbreviations: EMS, emergency medical services; NA, not applicable; OHCA, out-of-hospital cardiac arrest; OR, odds ratio.

^a^ Percentage change uses the following formula: 1 – (β excluding patients with acute infection / β all patients) or 1 – (β with Utstein covariates / β unadjusted excluding acute infection).

^b^ Refers to percentage change from total, unadjusted outcome.

^c^ Utstein elements for all EMS-treated patients with OHCA include location of arrest, witness status, automated external defibrillator use, EMS response interval (difference between time of 911 call to time of first unit at patient side), cause of arrest, bystander cardiopulmonary resuscitation, and OHCA before EMS arrival. Utstein elements for the Utstein group include location of arrest, automated external defibrillator use, EMS response interval (difference between time of 911 call to time of first unit at patient side), cause of arrest, and bystander cardiopulmonary resuscitation.

^d^ Refers to percentage change from unadjusted outcome excluding acute SARS-CoV-2 infection.

## Discussion

In this cohort investigation of COVID-19 and OHCA, we observed a 19.0% increase in OHCA (treated and untreated) incidence during the pandemic. Of all EMS-attended patients with OHCA, 6.2% of treated and 3.7% of untreated patients (approximately 5% overall) had evidence of acute SARS-CoV-2 infection. Temporal patterns of community-wide SARS-CoV-2 infection had modest correlation with OHCA incidence. OHCA survival was poorer during the pandemic years, a downturn largely attributable to Utstein elements, as opposed to more refractory resuscitation involving patients with acute SARS-CoV-2 infection. Collectively, the findings suggest that a substantial proportion of the increase in OHCA incidence and the decrease in OHCA survival was not due specifically to acute COVID-19, but rather indirect factors that more generally challenged OHCA prevention and treatment.

The current investigation leveraged a combination of data resources, including community COVID-19 surveillance, an established OHCA registry, and linkage between the registry and WDRS, to investigate how the pandemic and acute SARS-CoV-2 infection were associated with OHCA incidence and outcome. A better understanding of the predominant factors is important so that public health, clinical medicine, and emergency response can prioritize efforts. For example, excess OHCA incidence due directly to SARS-CoV-2 infection would direct resources to more effective prevention and treatment of COVID-19, whereas indirect pandemic factors would emphasize efforts supporting more general cardiovascular prevention and care and encourage persons with warning symptoms not to delay activating 911.

Overall, we observed a 27.2% increase in patients who were DOA and a 10.8% excess in EMS-treated patients with OHCA. The disproportionate increase among untreated patients with OHCA with irreversible death has been reported elsewhere^25^ and is perhaps expected, owing to less socialization during the pandemic^26,27,28^; opportunities to meaningfully engage in resuscitation rely on early recognition.^29^

COVID-19 can produce cardiac, pulmonary, and hematologic pathologies that could increase OHCA risk in the days and weeks following infection.^30,31^ However, approximately 5% of all patients with OHCA had evidence of acute SARS-CoV-2 infection, suggesting that acute pathophysiology of COVID-19 accounted for a small fraction of the incidence increase. Although SARS-CoV-2 infection could also directly affect OHCA risk over a protracted time course,^32,33^ the observed incidence increase was likely due to other pathways. Notably, the pandemic challenged multifaceted conventional cardiovascular prevention^34^ and discouraged and delayed 911 use because of fear of contracting SARS-CoV-2 during emergency care.^29,35,36^ One pandemic phenomenon that has been reported is a decline in acute ST-elevation myocardial infarction, perhaps related to patient reticence to seek emergency care.^25,37,38^ This behavior might translate to an increase in OHCA as disease progresses from ischemia to terminal arrhythmia resulting from treatment delays. Other epidemiologic forces could also contribute to increasing OHCA incidence, such as the opioid epidemic that has progressively claimed more lives in the US.^39^

OHCA incidence moderately corresponded to community-wide SARS-CoV-2 infection, typically trailing 1 to 2 weeks after an increase in community COVID-19. The association was particularly evident among patients with OHCA and acute SARS-CoV-2 infection and during 2020. One explanation for the differential correlation between 2020 and 2021 is the potential protective effects of vaccination against OHCA, given the high rate of vaccination among older adults in the study system.^21^

In the current study, OHCA survival was poorer during the pandemic compared with prior years, a finding that has been reported from other systems around the globe,^3,6,9,10,40,41^ likely translating to tens of thousands of excess deaths worldwide. The relative contribution of acute SARS-CoV-2 infection to OHCA survival decline has not been well-studied. We observed that 6.2% of treated patients during the pandemic period had acute SARS-CoV-2 infection, and 6.2% of acutely infected patients survived to discharge. Analyses indicate that acute infection was responsible for only 18.5% of the downturn in OHCA survival during the pandemic.

In contrast, the general profile of OHCA circumstances and prehospital care adversely changed during the pandemic period. Collectively, these changes in Utstein elements accounted for the majority of the pandemic survival decline, challenging both cardiac resuscitation and brain recovery. Fewer witnessed and public location OHCA events could be anticipated given social practices during the pandemic.^26,27,28^ The pandemic also produced changes in professional response and care. KCEMS practitioners added N-95 masks and gowns to eye protection and gloves as part of the updated personal protective equipment protocol for resuscitation.^17^ The approach delayed patient access by about a minute and translated to lower survival, highlighting the exquisitely time-sensitive nature of resuscitation. Conversely, the system did not adopt pandemic-invoked changes to telecommunicator coaching of bystander CPR, transition to mechanical CPR, or modify strategies of advanced airway management.^42^ The overarching goal was to retain practiced choreography that is integral in the teamwork required for successful resuscitation.^42^ We were also buoyed by hospital postresuscitation efforts to continue interventional coronary evaluation and temperature management, although there was evidence that the relative timing of these interventions changed between prepandemic and pandemic periods.

### Limitations

The study has limitations. The data are from a regional EMS system that serves a large, populous county. This region was the starting point for the COVID-19 pandemic in the US but subsequently had an effective community-wide effort to prevent and manage SARS-CoV-2 infection.^43^ Systems with distinct COVID-19 epidemiology or EMS response may have different experiences, although the fidelity of OHCA ascertainment and COVID-19 classification enables a useful assessment of how COVID-19 and the pandemic period affected OHCA incidence and outcome. Demographic, clinical, and identifying information were incomplete among DOA patients, limiting the ability to classify COVID-19 status and undertake stratified analyses involving this group. Classification of acute infection incorporated testing results and clinical information, although testing was not comprehensive. To the extent that those without testing may not have reported common COVID-19 symptoms, the approach may have underestimated the prevalence of acute infection and could explain the lower proportion among DOA patients. Although we classified patients with OHCA according to acute SARS-CoV-2 infection, we cannot determine whether the virus caused OHCA or was simply present during the arrest. Only 194 treated patients with OHCA had acute SARS-CoV-2 infection, potentially limiting power.

## Conclusions

In this cohort study of COVID-19 and OHCA, we observed higher OHCA incidence during the pandemic, although patients with acute SARS-CoV-2 infection composed a small fraction of OHCA cases. OHCA survival was poorer during the pandemic years, largely owing to changes in systemwide Utstein characteristics, as opposed to patient-specific acute SARS-CoV-2 infection. Overall, the findings underscore the adverse public health consequences from indirect, sometimes unanticipated, effects of the pandemic.

## Notes

Supplement 1. **eFigure 1.** Flow Diagram of Acute SARS-CoV-2 Infection Classification for EMS-Treated Out-of-Hospital Cardiac Arrest **eFigure 2.** Flow Diagram of Acute SARS-CoV-2 Infection Classification for EMS-Attended Dead-On-Arrival Out-of-Hospital Cardiac Arrest **eFigure 3.** Directed Acyclic Graph of Association Between Time Period and Resuscitation Outcomes **eFigure 4.** Trend of Weekly (Lighter Color) and Four Week Moving Average (Darker Color) of COVID-19 and EMS-Treated Out-of-Hospital Cardiac Arrest Incidence per 100,000 **eTable.** Characteristics of EMS-Treated Out-of-Hospital Cardiac Arrest (OHCA) of the Utstein Subgroup (Bystander Witnessed With Initial Shockable Rhythm) According to Time Period and SARS-CoV-2 Status

Supplement 2.
Data Sharing Statement
