# Supplementary material for: Acute SARS-CoV-2 Infection and Incidence and Outcomes of Out-of-Hospital Cardiac Arrest
Source: JAMA Netw Open. 2023 Oct 6;6(10):e2336992. doi: 10.1001/jamanetworkopen.2023.36992 (PMC10559182; doi:10.1001/jamanetworkopen.2023.36992)
Supplement: Supplement 1. — eFigure 1. Flow Diagram of Acute SARS-CoV-2 Infection Classification for EMS-Treated Out-of-Hospital Cardiac Arrest eFigure 2. Flow Diagram of Acute SARS-CoV-2 Infection Classification for EMS-Attended Dead-On-Arrival Out-of-Hospital Cardiac Arrest eFigure 3. Directed Acyclic Graph of Association Between Time Period and Resuscitation Outcomes eFigure 4. Trend of Weekly (Lighter Color) and Four Week Moving Average (Darker Color) of COVID-19 and EMS-Treated Out-of-Hospital Cardiac Arrest Incidence per 100,000 eTable. Characteristics of EMS-Treated Out-of-Hospital Cardiac Arrest (OHCA) of the Utstein Subgroup (Bystander Witnessed With Initial Shockable Rhythm) According to Time Period and SARS-CoV-2 Status [file jamanetwopen-e2336992-s001.pdf]

Supplemental Online Content

Liu JZ, Counts CR, Drucker CJ, et al. Acute SARS-CoV-2 infection and incidence and outcomes of out-of-hospital cardiac arrest. *JAMA Netw Open*. 2023;6(10):e2336992. doi:10.1001/jamanetworkopen.2023.36992

eFigure 1. Flow Diagram of Acute SARS-CoV-2 Infection Classification for EMS-Treated Out-of-Hospital Cardiac Arrest

eFigure 2. Flow Diagram of Acute SARS-CoV-2 Infection Classification for EMS-Attended Dead-On-Arrival Out-of-Hospital Cardiac Arrest

eFigure 3. Directed Acyclic Graph of Association Between Time Period and Resuscitation Outcomes

eFigure 4. Trend of Weekly (Lighter Color) and Four Week Moving Average (Darker Color) of COVID-19 and EMS-Treated Out-of-Hospital Cardiac Arrest Incidence per 100,000

eTable. Characteristics of EMS-Treated Out-of-Hospital Cardiac Arrest (OHCA) of the Utstein Subgroup (Bystander Witnessed With Initial Shockable Rhythm) According to Time Period and SARS-CoV-2 Status

This supplemental material has been provided by the authors to give readers additional information about their work.

© 2023 Liu JZ et al. *JAMA Network Open.*

eFigure 1. Flow Diagram of Acute SARS-CoV-2 Infection Classification for EMS-Treated Out-of-Hospital Cardiac Arrest

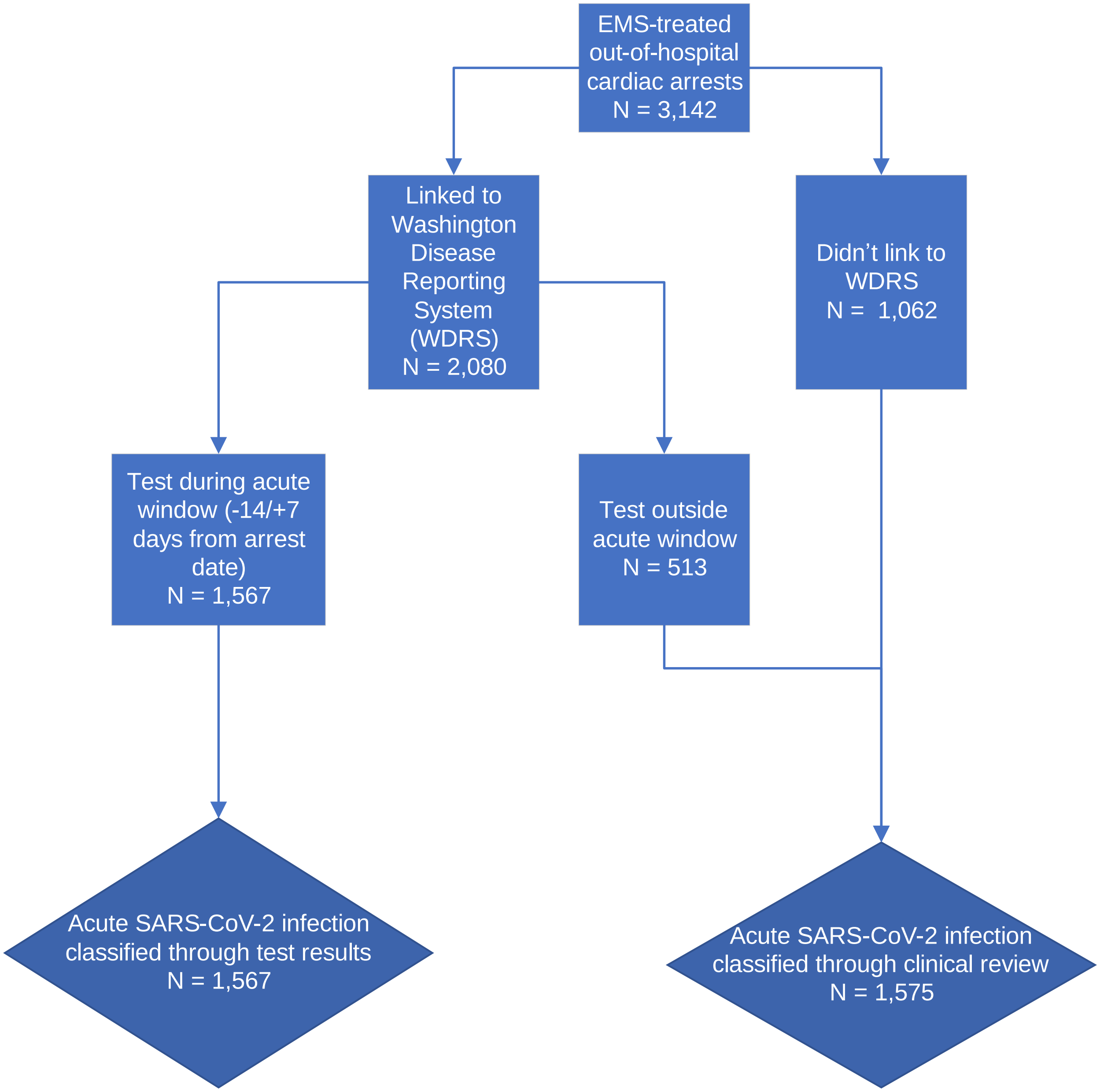

Abbreviations: EMS, emergency medical services.
Notes: WDRS contains name, date of birth, SARS-CoV-2 test dates, and SARS-CoV-2 results for all individuals who have been tested in Washington State.

© 2023 Liu JZ et al. *JAMA Network Open.*

eFigure 2. Flow Diagram of Acute SARS-CoV-2 Infection Classification for EMS-Attended Dead-On-Arrival Out-of-Hospital Cardiac Arrest

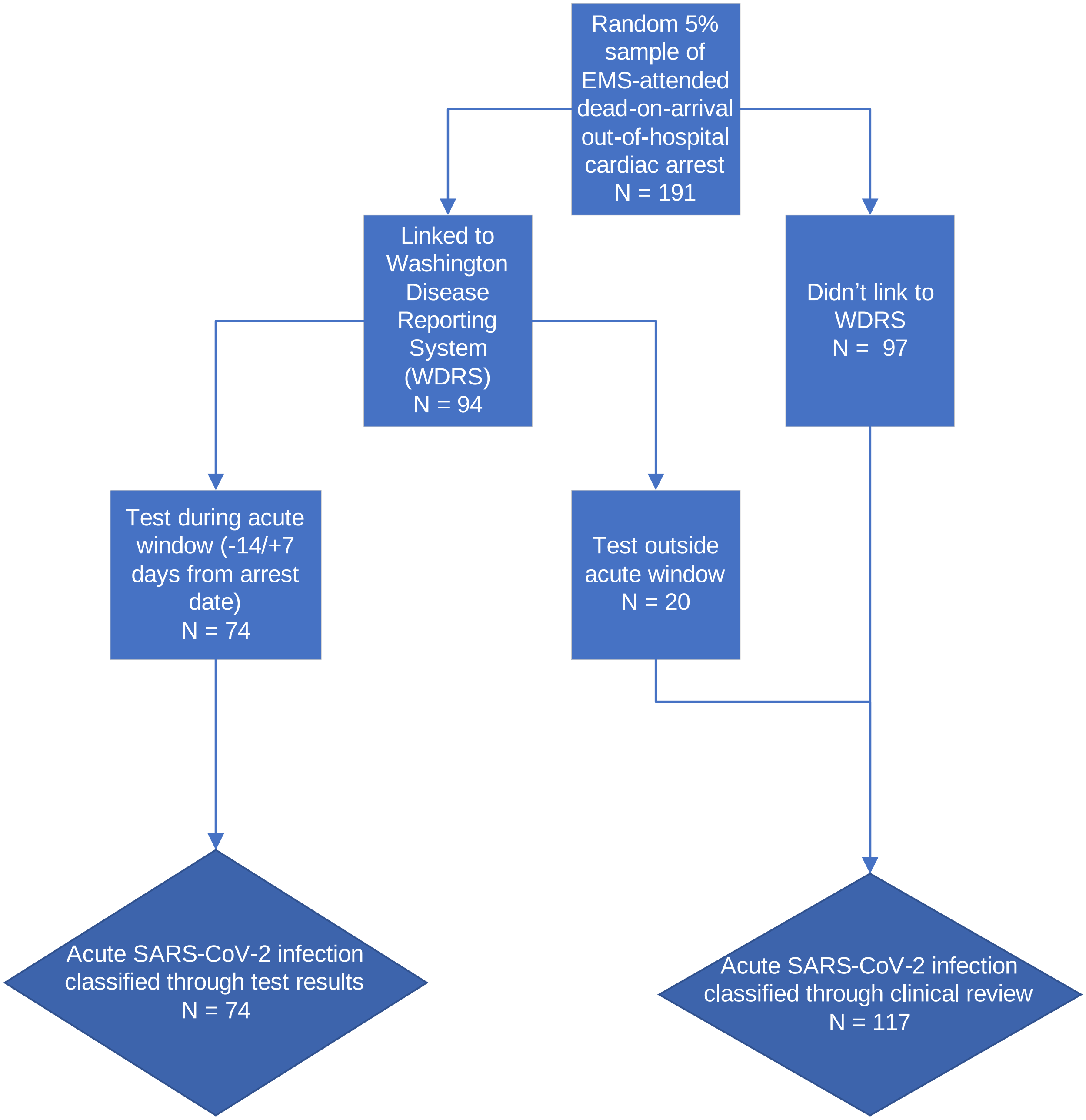

Abbreviations: EMS, emergency medical services.
Notes: WDRS contains name, date of birth, SARS-CoV-2 test dates, and SARS-CoV-2 results for all individuals who have been tested in Washington State.

© 2023 Liu JZ et al. *JAMA Network Open.*

eFigure 3. Directed Acyclic Graph of Association Between Time Period and Resuscitation Outcomes

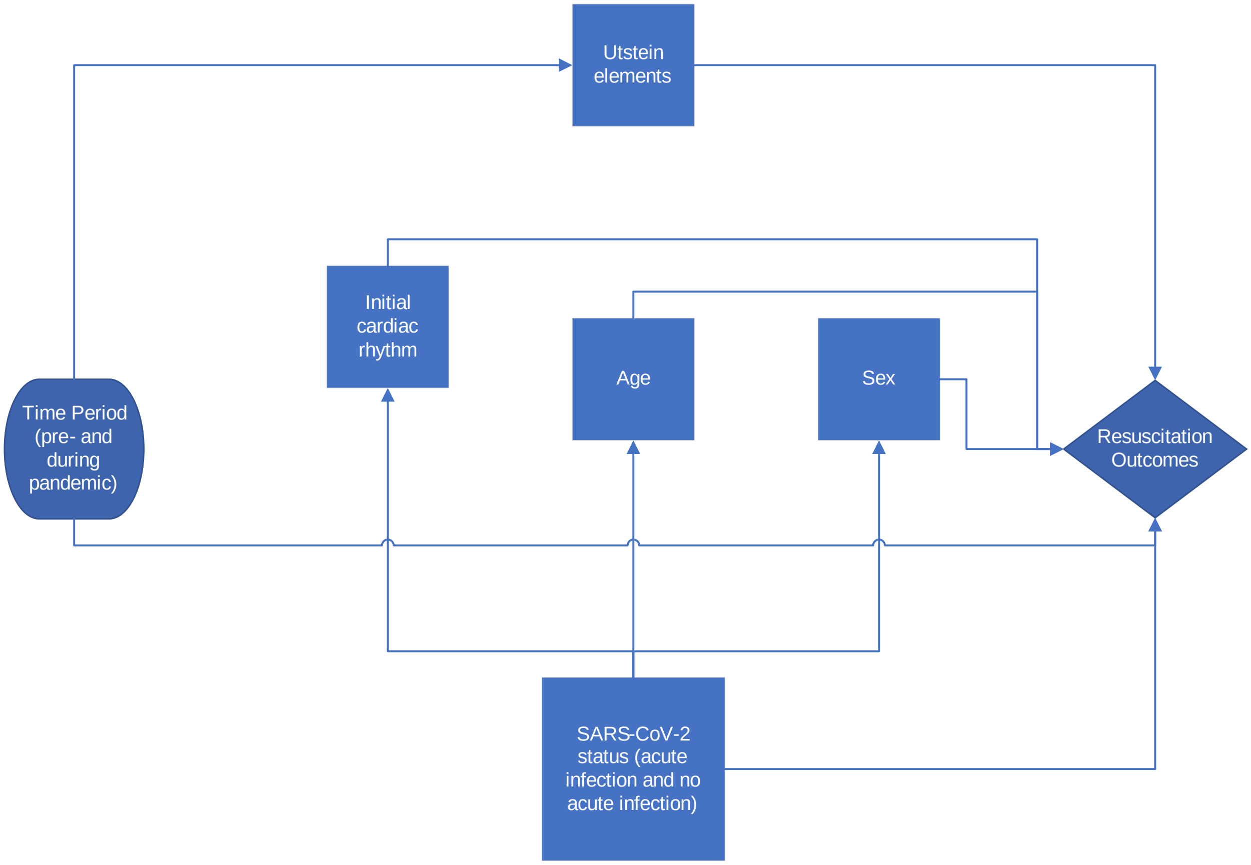

Notes: Utstein elements include etiology of arrest, arrest location, witness status, bystander cardiopulmonary resuscitation, non-emergency medical services (EMS) automated external defibrillator application, response interval from 9-1-1 call to EMS at-patient's-side, and arrest before EMS arrival. Exposure is time period and outcome is resuscitation outcomes.

© 2023 Liu JZ et al. *JAMA Network Open.*

eFigure 4. Trend of Weekly (Lighter Color) and Four Week Moving Average (Darker Color) of COVID-19 and EMS-Treated Out-of-Hospital Cardiac Arrest Incidence per 100,000.

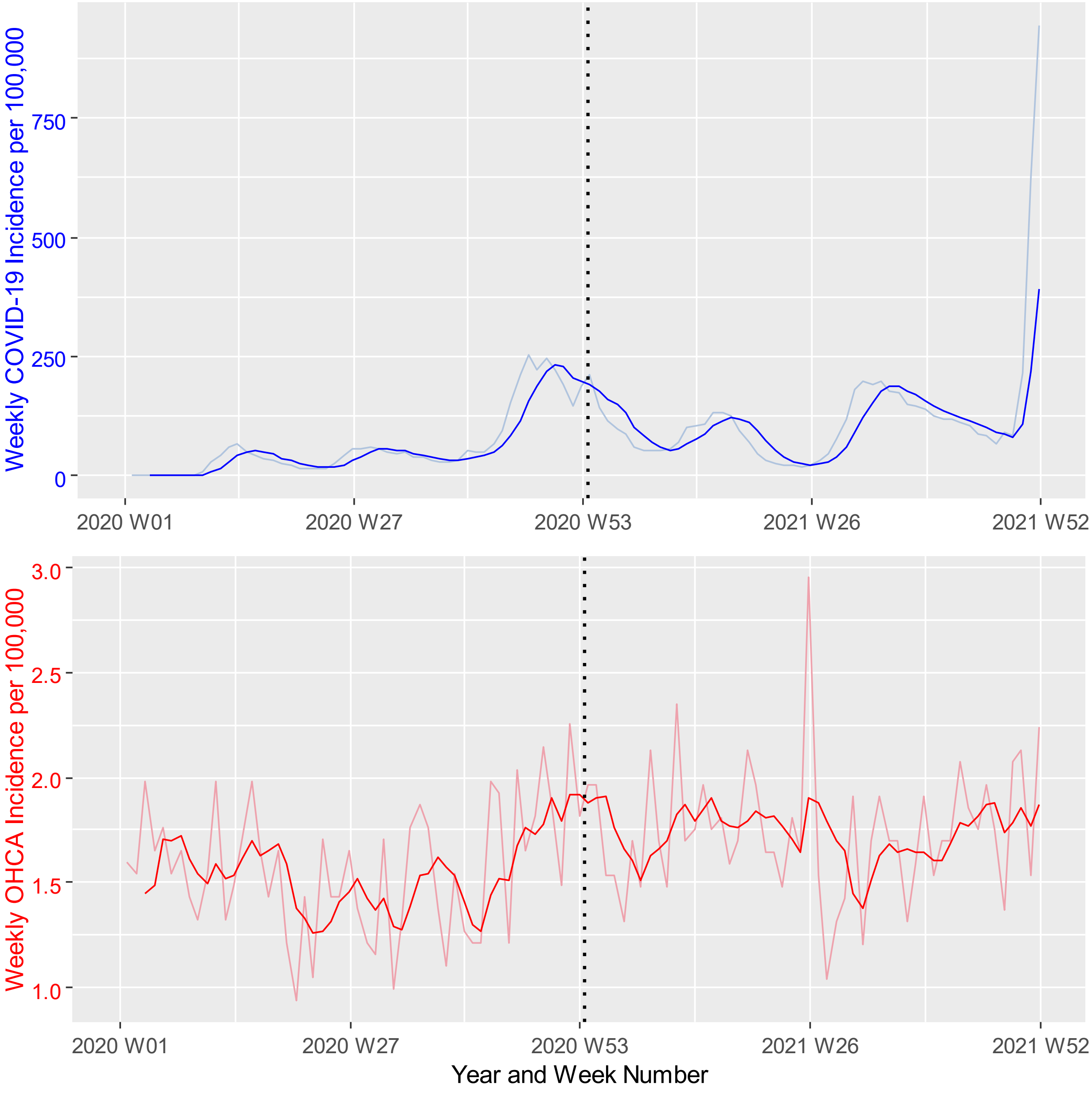

Abbreviations: COVID-19, Coronavirus Disease 2019; OHCA, Out-of-Hospital Cardiac Arrest.
Notes: Cross correlation coefficient was 0.27. Lighter color represents weekly incidence and darker color represents four week moving average. Vertical black dotted line represents the separate years, with year 2020 on the left and year 2021 on the right.

© 2023 Liu JZ et al. *JAMA Network Open.*

eTable. Characteristics of EMS-Treated Out-of-Hospital Cardiac Arrest (OHCA) of the Utstein Subgroup (Bystander Witnessed With Initial Shockable Rhythm) According to Time Period and SARS-CoV-2 Status

				Pandemic Period		
	**Pre-pandemic Period[a] (n = 428)**	**Pandemic Period[a] (n = 421)**	***P* Value**	**No Acute SARS-CoV-2 Infection[a] (n = 410)**	**Acute SARS-CoV-2 Infection[a] (n = 11)**	***P* Value**
Age, median (IQR), y	63 (53.0-71.0)	64 (53.0-73.0)	0.56	64 (53.0-73.0)	57 (51.0-65.0)	0.36
Male, No. (%)	88 (20.6)	93 (22.1)	0.65	91 (22.2)	2 (18.2)	0.99
Cardiac Etiology, No. (%)	395 (92.3)	382 (90.7)	0.68	373 (91.0)	9 (81.8)	0.27
Location, No. (%)						
Home/Other Residence	209 (48.8)	266 (63.2)	<0.001	259 (63.2)	7 (63.6)	0.79
Public (Indoors, Outdoors)	194 (45.3)	125 (29.7)		122 (29.8)	3 (27.3)	
Care Facility (long-term care, clinic)	25 (5.84)	30 (7.1)		29 (7.1)	1 (9.1)	
Bystander CPR[b], No. (%)	356 (83.2)	351 (83.4)	0.99	341 (83.2)	10 (90.9)	0.70
Non-EMS AED Application[b], No. (%)	82 (19.2)	64 (15.2)		61 (14.9)	3 (27.3)	
Law Enforcement	35 (8.2)	30 (7.1)	0.27	29 (7.1)	1 (9.1)	0.23
Layperson	47 (11.0)	34 (8.1)		32 (7.8)	2 (18.2)	
9-1-1 to BLS Scene Arrival, median (IQR), min	4.5 (3.6-5.8)	5.1 (3.9-6.3)	<0.001	5.1 (3.9-6.3)	5.3 (4.9-5.9)	0.36
9-1-1 to ALS scene Arrival, median (IQR), min	7.5 (5.3-9.8)	8.0 (6.1-10.1)	0.02	8.0 (6.1-10.0)	9.7 (8.5-10.6)	0.08
9-1-1 to EMS-at-patient's-side Interval, median (IQR), min	6.5 (5.4-7.9)	7.5 (5.9-7.5)	<0.001	7.4 (5.9-9.0)	7.6 (6.1-9.0)	0.94
Admitted to Hospital, No. (%)	327 (76.4)	282 (67.0)	0.003	277 (67.6)	5 (45.5)	0.19
Hospital Care[c]						
Targeted Temperature Management, No. (%)	218 (66.7)	214 (75.9)	0.03	211 (76.2)	3 (60.0)	0.60
Angiogram, No. (%)	265 (81.0)	210 (74.5)	0.06	206 (74.4)	4 (80.0)	0.99
Time from 911 Call to Angiogram, No. (%)						
<3 hours	146 (55.1)	98 (46.7)	0.10	97 (47.1)	1 (25.0)	0.36
<6 hours	165 (62.3)	115 (54.8)	0.15	114 (55.3)	1 (25.0)	0.30
<24 hours	189 (71.3)	128 (61.0)	0.02	126 (61.2)	2 (50.0)	0.60

Abbreviations: OHCA, out-of-hospital cardiac arrest; EMS, emergency medical services; CPR, cardiopulmonary resuscitation; AED, automated external defibrillator; BLS, basic life support; ALS, advanced life support.

[a]Pre-pandemic period includes years 2018-2019, and pandemic period includes years 2020-2021. Acute SARS-CoV-2 infection status is only during the pandemic period.

[b]Restricted to OHCAs before EMS arrival.

[c]Restricted to patients admitted to the hospital.

© 2023 Liu JZ et al. *JAMA Network Open.*

eTable (cont). Characteristics of EMS-Treated Out-of-Hospital Cardiac Arrest (OHCA) of the Utstein Subgroup (Bystander Witnessed With Initial Shockable Rhythm) According to Time Period and SARS-CoV-2 Status

				Pandemic Period		
	**Pre-pandemic Period[a] (n = 428)**	**Pandemic Period[a] (n = 421)**	***P* Value**	**No Acute SARS-CoV-2 Infection[a] (n = 410)**	**Acute SARS-CoV-2 Infection[a] (n = 11)**	***P* Value**
Withdrawal of Life Sustaining Treatment, No. (%)	79 (24.2)	86 (30.5)	0.09	85 (30.7)	1 (20.0)	0.99
Time from Hospital Admission to Withdrawal of Care, median (IQR), d	4 (1.0-6.0)	3 (1.0-7.3)	0.79	3 (1.0-7.5)	4 (4.0-4.0)	0.85
Survival, No. (%)						
Survived to Hospital Discharge	234 (54.7)	176 (41.8)	<0.001	172 (42.0)	4 (36.4)	0.77
Favorable Neurological Survival (CPC 1-2)	216 (50.5)	163 (38.7)	<0.001	159 (38.8)	4 (36.4)	0.99

Abbreviations: OHCA, out-of-hospital cardiac arrest; EMS, emergency medical services; CPR, cardiopulmonary resuscitation; AED, automated external defibrillator; BLS, basic life support; ALS, advanced life support; CPC, cerebral performance category.

[a]Pre-pandemic period includes years 2018-2019, and pandemic period includes years 2020-2021. Acute SARS-CoV-2 infection status is only during the pandemic period.

[b]Restricted to OHCAs before EMS arrival.

[c]Restricted to patients admitted to the hospital.

© 2023 Liu JZ et al. *JAMA Network Open.*
