# Supplementary material for: Acute SARS-CoV-2 Infection and Incidence and Outcomes of Out-of-Hospital Cardiac Arrest
Source: JAMA Netw Open. 2023 Oct 6;6(10):e2336992. doi: 10.1001/jamanetworkopen.2023.36992 (PMC10559182; doi:10.1001/jamanetworkopen.2023.36992)
Supplement: Supplement 2. — Data Sharing Statement [file jamanetwopen-e2336992-s002.pdf]

Data Sharing Statement

Liu. Acute SARS-CoV-2 Infection and Incidence and Outcomes of Out-of-Hospital Cardiac Arrest. *JAMA Netw Open*. Published October 06, 2023.
doi:10.1001/jamanetworkopen.2023.36992

Data
Data available: No
